# Low-Cost Bicycle Lights vs. Cold Lights for Visualizing Neonatal Veins

**DOI:** 10.1093/tropej/fmx049

**Published:** 2017-08-10

**Authors:** Neal J Russell, Paul Bassett, John Chang

**Affiliations:** 1 London School of Hygiene & Tropical Medicine, London WC1E 7HT, UK; 2 Statsconsultancy Ltd, Amersham, Bucks HP7 9EN, UK; 3 Croydon Research Department, Croydon University Hospital, Croydon, Surrey CR7 7YE, UK

**Keywords:** difficult intravenous access, intravenous cannulation, venepuncture, neonatal, transillumination

## Abstract

**Objective:**

Difficult intravenous (IV) access can compromise patient care in neonatal practice, and transillumination is often used to improve the visibility of veins. Current devices are expensive, prone to bacterial contamination and unaffordable in low-resource settings. We conducted a study comparing the quality of transillumination provided by "cold lights" that are currently in use with low-cost (<£1) red silicone LED bicycle lights.

**Methods:**

Photographs of the hands and feet of neonates were taken with parental consent: first without transillumination (control group), second by transillumination with a cold light, and third with a bicycle light. Thirty photographs were sent in a survey to pediatric doctors who were blinded to the method of transillumination. Survey respondents then rated the visibility of the veins (easily visible, moderately visible, barely visible and invisible).

**Results:**

Completed surveys of 114 respondents were included in the analysis. The majority (94.8%) of respondents rated the veins moderately to easily visible with the bicycle light compared with 87.6% with the cold light, and 42.6% in the control group with no transillumination. There was a strong evidence of an improvement in visibility with bicycle lights compared with cold lights (*p* < 0.001).

**Conclusion:**

Low-cost red silicone LED bicycle lights were found to improve visibility of veins in neonates. Given their quality of transillumination, portability and reduced cost, they may provide a useful method of transillumination in all settings, but particularly in low-income settings, where there is currently no affordable alternative.

## BACKGROUND

Difficult intravenous (IV) access is a long-standing and familiar problem in neonatology and pediatrics, and can significantly compromise clinical care in some circumstances. Transillumination has been recognized as a useful technique in overcoming this challenge for some time [1–5]. However, the devices most commonly used for transillumination (cold lights) are prone to bacterial contamination, can be difficult to use in a clean or sterile manner, and are expensive. Their risk of cross-infection was highlighted recently in an unpublished study showing the cold light to be the most heavily contaminated piece of equipment on our neonatal unit, for example (L. Young, Unpublished) [6]. Given their cost, cold lights are often shared among many babies, and could therefore be a vector of hospital-acquired infections (similar to stethoscopes). New methods such as infra-red devices are being developed to overcome these issues, but are even more expensive and their effectiveness has not been consistently demonstrated [7, 8, 12–14]. Ultrasound is emerging as a useful method for obtaining longer term IV access in deeper veins, but is also expensive and less useful for standard peripheral IV access in superficial veins in neonates [9]. Therefore, there is a need for low-cost, user-friendly and safe devices with minimal infection risk—particularly in low-income countries where the majority of neonatal deaths occur [10], and where all currently available transillumination devices are unaffordable.

Red silicone LED bicycle lights, which have anecdotally been used for IV cannulation and venepuncture, as well as arterial access and peripherally inserted central lines, are a possible low-cost alternative method for transillumination. At a cost of less than £1 each, compared to £100s–1000s for most of the other devices on the market, their economic advantage is clear. They are also easily portable, and owing to their small size can be completely enclosed inside clinical or sterile gloves (unlike most cold lights), reducing the risk of transmission of infection.

In terms of safety, the red silicone LED bicycle lights used in this study use similar LED technology to many of the cold lights currently in use. Formal testing locally demonstrated no rise in temperature with use, and a power output of ∼226 mW/cm^2^, much less than other devices used on neonates such as cranial ultrasounds whose output is ∼800 mW/cm^2^. In terms of radiation emission, although there is no accepted limit, there should be no safety concerns with bicycle lights used for short periods given that use of lasers with similar power outputs for up to 8 h is considered safe [11]. A similar time period to that recommended for cold lights (up to 4 min) would be reasonable. Furthermore, purely red lights are thought to be safer than white lights which are often used in cold lights [11].

To our knowledge, there are no published studies looking at low-cost methods of transillumination, as most research has been driven by new development of expensive medical devices. We set out to test the quality of transillumination provided by low-cost red silicone LED bicycle lights in neonates by comparing them with a standard cold light currently in use.

## METHODS

An online survey comparing the visibility of bicycle lights and cold lights was conducted. Neonates in the Neonatal Unit at Croydon University Hospital were selected opportunistically between December 2014 and January 2015. Babies who were clinically unstable or whose parents did not provide consent were excluded.

**Fig. 1.**
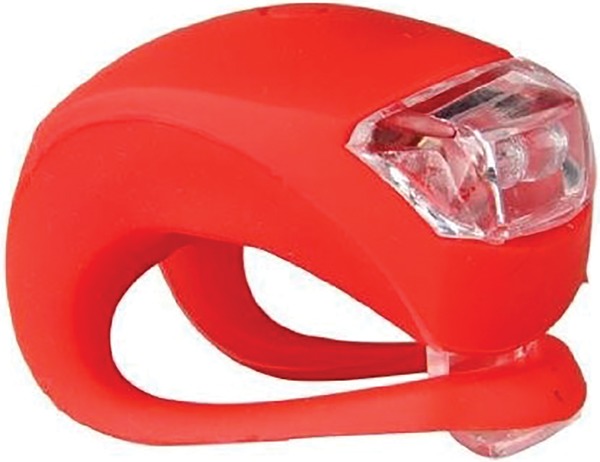
An example of a red silicone LED bicycle light (available from many different manufacturers).

**Fig. 2.**
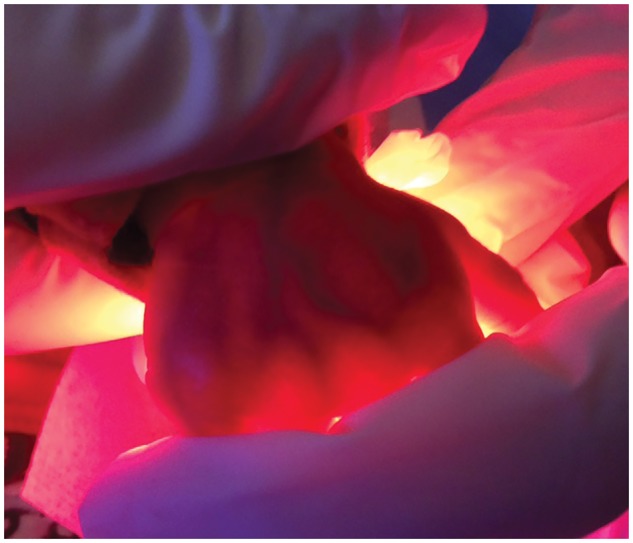
Example of transillumination with an LED bicycle light.

Sets of three photographs were taken of hands and feet (i) with no transillumination (control), (ii) with transillumination with a cold light, and (iii) with a red silicone LED bicycle light (Figs 1 and 2). Photos were taken in the Neonatal Intensive Care and Special Care Baby Unit in the normal clinical environment. Babies remained in their incubators, the background lighting being dimmed to the same level for the cold lights and bicycle lights, and kept at normal room lighting for photos without transillumination. The bicycle lights were enclosed in a clinical glove, which is how we propose they should be used in practice to reduce infection risk.

Photographs were taken by a team of two researchers with an iPhone 4s. One researcher held the hand or foot of the baby, without a tourniquet, and the other took the photographs. Strict infection control procedures were adhered to throughout. The best quality photograph was selected among several of the same limb and transillumination approach. Sixty photos of 20 hands or feet (with a control, cold light and red LED light picture of each) were used in a pilot survey with 12 independent respondents. Based on respondents’ feedback, the number of photos was cut down to 30 (10 hands or feet from five babies) after removing pictures of lower quality.

We designed an online survey including 10 sets of three photographs (one control, one cold light and one bike light) from each hand or foot. The best quality photographs from each limb were chosen to limit variation in quality. All photographs were reshuffled randomly for each respondent, who were asked to rate the ease of visibility of veins for intravenous cannulation (invisible, barely visible, moderately visible or easily visible). Respondents were blinded both to the method of transillumination in the photographs, as well as to the purpose of the study.

For statistical analysis, the data were considered to be clustered and non-independent at the levels of the observers and individual body parts, as a given observer may rate photographs in a certain way, and there are likely to be similarities in the way observers rate particular body parts. A multilevel logistic regression model with random effects was carried out, with a cross-classified structure for both body part and observer. Ease of visibility (the outcome variable) was categorized into a binary variable with categories Invisible/Barely visible and Moderately/Easily visible. The multilevel regression model also included skin colour (black or white) as an explanatory variable. We tested the hypothesis that the effect of bicycle light on visibility would differ by skin colour by examining the *p*-value for an interaction term between skin colour and type of light. Statistical analyses were carried out using Stata Version 13.1.

This survey was conducted within the context of a randomized controlled trial comparing the use of cold lights and bicycle lights for intravenous cannulation, which received ethical approval from the NHS Research Ethics Committee (Ref: 14/LO/1723). Screening on the same IRAS system provided written confirmation that separate ethical approval was not required. Written consent was obtained from parents after being given an information leaflet, and given the opportunity to ask questions. It was ensured that no photographs contained identifiable information on any of the babies.

## RESULTS

Ten sets of three photographs (control, cold light and bicycle light) of hands or feet from five neonates were included in the final survey. The characteristics of these five babies are presented in Table 1. The online survey was sent by email to 281 pediatric doctors in training in the south London deanery, of whom 128 responded. Fourteen questionnaires were excluded owing to missing responses for ≥40% questions.

**Table 1:** Characteristics of babies included in final survey

	Gestational Age at Birth	Corrected Gestational Age	Current Weight (g)	Ethnicity
Baby 1	28+2	32+5	1374	Afro-Caribbean
Baby 2	28+2	32+5	1330	Afro-Caribbean
Baby 3	28+5	37+3	2510	Afro-Caribbean
Baby 4	36+3	36+6	1774	Caucasian
Baby 5	30+0	34+1	2096	Caucasian

**Chart 1:**
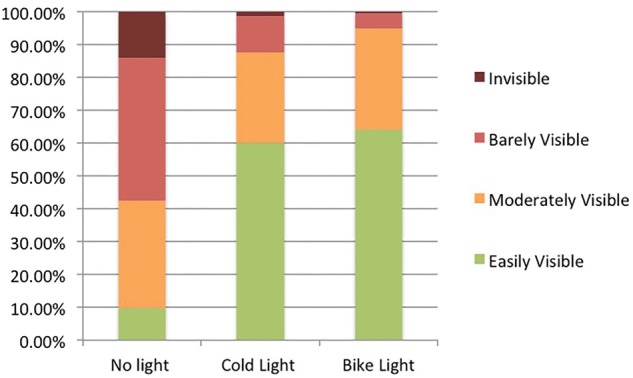
Visibility of veins by method of transillumination.

As shown in Table 2 and Chart 1, among the 114 responses analysed, respondents considered the veins of babies to be moderately or easily visible in 94.8% of cases when illuminated with bicycle light, compared with 87.6% with cold light, and 42.6% of cases in the control group without transillumination.

**Table 2:** Responses by light source in study

	Invisible	Barely Visible	Moderately Visible	Easily Visible	Total Responses
No light	162 (14.2%)	492 (43.3%)	369 (32.7%)	113 (9.9%)	1136
Cold light	15 (1.3%)	126 (11.1%)	312 (27.6%)	679 (60%)	1132
Bike light	5 (0.4%)	54 (4.8%)	347 (30.6%)	728 (64.2%)	1134

The multilevel logistic regression model (shown in Table 3) demonstrated that, compared with no light, the odds of veins being moderately to easily visible were higher for both cold light [OR 12.5 (9.92–15.8)] and bicycle light [OR 34.1 (25.1–46.3)]. The bicycle light also performed better than the cold light [OR 2.72 (1.97–3.76)].

**Table 3:** Odds of moderate/easy visibility by light source: multilevel regression

Variable	Comparison	Odds Ratio	*p*-value
Light type	Cold light vs. None	12.5 (9.92, 15.8)	<0.001
	Bike light vs. None	34.1 (25.1, 46.3)	<0.001
	Bike light vs. Cold light	2.72 (1.97, 3.76)	<0.001
Skin colour	Black/White	1.17 (0.42, 3.22)	0.76

Note: Using a cross-classified structure, with random effects for both participant and body part.

There was no evidence of an interaction between type of light and skin colour (*p*-value for the interaction term = 0.13), and neither did skin colour affect the visibility of veins in our sample (OR 1.17, 0.42–3.22). There were no adverse events in any of the subjects in the study.

## DISCUSSION

This study demonstrates that the quality of transillumination with low-cost red silicone LED bicycle lights is at least equivalent to that of standard cold lights, and may be better. Two-thirds (64%) of doctors in our sample judged neonates’ veins to be easily visible with bicycle lights, and a further 31% moderately visible (compared with 60% and 28% for cold lights, respectively). These results suggest that LED bicycle lights are a good alternative to standard cold lights.

There are several potential justifications for using these bicycle lights for IV cannulation of neonates. First, their low cost (<£1) provides a strong economic argument even without their observed improved transillumination over standard cold lights, and may allow for increased availability of transillumination devices on the wards (perhaps even for single use). Second, even without considering single-use, the ability to completely enclose the bicycle lights inside gloves reduces the risk of cross-infection compared with cold lights, many of which cannot be enclosed in such a way. The reality is that in a neonatal unit, owing to their cost, one or two cold lights are shared amongst several babies, raising concerns given their susceptibility to bacterial contamination. This situation is reminiscent of the concerns with stethoscopes, which were likely acting as vectors of hospital-acquired infections before the policy of ‘one stethoscope per baby’ was adopted. Therefore, the use of red LED bicycle lights (whether single-use or enclosed within gloves) has the potential to prompt a shift in practice which could reduce this cross-infection risk. Aside from these advantages in settings where cold lights are already available, red LED bicycle lights represent a possible low-cost option for transillumination in low-resource setting where there are no other affordable options. In theory, any red LED light which is affordable and of appropriate size could be used for this purpose.

The results of the study are strengthened by the large number of respondents (114) each rating the visibility of veins in 30 photographs with few missing data (0.5% missing answers in analysed responses), all of whom were pediatric doctors who regularly cannulate neonates. Bias was minimized by selecting babies without prior examination of their veins, and by blinding participants to the method of transillumination and to the purpose of the study. The sequence of individual photographs was randomized (across babies and methods), to minimize any potential bias related to order of appearance.

Limitations of the study included variations in picture quality; however, this was minimized by taking large numbers of photographs (187) on the same camera, from which the 30 best quality photos were selected, and included photographs were from the same camera, affecting each group equally. Bias in photograph selection was minimized by selecting these photographs based on image quality rather than transillumination quality and on assessments of 60 photographs from independent doctors not involved in the study. Owing to the potential for respondent attrition in long surveys, it was decided to restrict the number of photographs to 30 to increase completeness of responses. This was of particular importance given the random order of the questionnaires, and need for all three photograph categories from each body part to be rated by each respondent to allow comparison of techniques.

This study included limited variation in age (32–37 weeks corrected), with no data on other ages, but it could be expected as with cold lights that bicycle lights could be used in older children depending on hand or foot width.

## CONCLUSION

Red silicone LED bicycle lights, costing <£1, are at least as effective as standard cold light sources in producing high quality transillumination of veins in neonates. Given the obvious economic advantages, the likely reduced risk of cross-infection with their use inside gloves, and their portability, they should be considered for use in neonates. Randomized controlled trial evidence comparing success rate of cannulation between cold lights and red LED bicycle lights would be useful in assessing further the effectiveness of these lights. However, in settings where no other transillumination devices are available, clinicians should already consider using a red LED bicycle light when faced with a baby with difficult veins.

WHAT IS ALREADY KNOWN ON THIS TOPIC?

- Transillumination is a useful technique for difficult intravenous cannulation, particularly in neonates
- Current devices are expensive, unaffordable in low-resource settings

WHAT THIS STUDY ADDS

- Red silicone LED bicycle lights, costing <£1, are at least as good as cold lights for improving the visibility of veins in neonates
- These bicycle lights could be used in low-resource settings where alternative methods are unaffordable
- Where cold lights are available, red LED bicycle lights may be an alternative method of transillumination with several clinical advantages such as lower risk of cross-infection

## WHAT IS ALREADY KNOWN ON THIS TOPIC?

- Transillumination is a useful technique for difficult intravenous cannulation, particularly in neonates
- Current devices are expensive, unaffordable in low-resource settings

## WHAT THIS STUDY ADDS

- Red silicone LED bicycle lights, costing <£1, are at least as good as cold lights for improving the visibility of veins in neonates
- These bicycle lights could be used in low-resource settings where alternative methods are unaffordable
- Where cold lights are available, red LED bicycle lights may be an alternative method of transillumination with several clinical advantages such as lower risk of cross-infection
